# Capacity building in European health information systems: the InfAct peer assessment methodology

**DOI:** 10.1093/eurpub/ckac014

**Published:** 2022-03-23

**Authors:** Petronille Bogaert, Marieke Verschuuren, Herman Van Oyen, Hans van Oers

**Affiliations:** Department of Epidemiology and Public Health, Sciensano, Belgium; Tilburg School of Social and Behavioral Sciences, Tilburg University, The Netherlands; Independent Public Health Consultant, Utrecht, The Netherlands; Department of Epidemiology and Public Health, Sciensano, Belgium; Department of Public Health, Ghent University, Belgium; Tilburg School of Social and Behavioral Sciences, Tilburg University, The Netherlands

## Abstract

**Background:**

A Health Information System (HIS) assessment is an evaluation of the functioning of the main elements that compose a national HIS. Assessors from nine countries performed peer assessments of each other’s national HIS in the Joint Action on Health Information (InfAct). The aim of this study is to evaluate the advantages and disadvantages of the InfAct peer assessment methodology as well as the different steps involved in this assessment process.

**Methods:**

Each peer assessment included a preparatory desk report, a country visit with semi-structured interviews with local stakeholders, a final report and a follow-up stakeholder meeting. A qualitative content analysis of the peer HIS assessment was performed based on 12 semi-structured interviews.

**Results:**

The main advantage of the assessments is its informal atmosphere, high degree of objectiveness and its networking opportunities. Disadvantages are its informal request format and setting for recommendation uptake. The peer assessment helped the assessors to broaden their understanding of the assessed and their own HISs, to gain knowledge on how to carry out an HIS assessment and to practice their organization, communication, reporting and negotiation skills. All steps of the HIS assessment are essential and each contributes to the enriching experience of the participants.

**Conclusion:**

The InfAct peer HIS assessment methodology strengthened capacity in national HISs by building up the knowledge and expertise in participating countries and as such addressed health information inequalities. This study confirms the value and relatively easy to implement methodology, and therefore recommends its wide and more systematic application across Europe.

## Introduction

A Health Information System (HIS) assessment is an evaluation of the functioning of the main elements that compose a national HIS. HIS elements include data collection, analysis and synthesis, reporting, and knowledge translation, and the total of resources, stakeholders, activities and outputs to do so.^1^ The purpose of an HIS assessment is to identify strengths and weaknesses in the HIS and to stimulate actions for improvement.

Several international organizations have developed well-established tools for HIS assessments. The World Health Organization (WHO) Regional Office for Europe *Support tool to assess HISs and develop and strengthen health information strategies* is one of these.^2–6^ This tool applies a comprehensive approach and captures various aspects of HISs, such as governance, databases, indicators and resources. Another example is the *HIS Stages of Continuous Improvement Toolkit* developed by MEASURE.^7^ It was designed to help countries or organizations holistically assess, plan and prioritize interventions and investments to strengthen a HIS. Other tools are the *Information Systems for Health Standard Assessment Method* of Pan-American Health Organization and the WHO *SCORE for Health Data Technical Package*.^8^^,^^9^ HIS assessments can be self-administered, externally administered, peer administered or have a hybrid methodology. Peer administered means the HIS assessment is performed by one or more expert representatives of a different HIS than the one under assessment.

One of the goals of the EU Joint Action (JA) on Health Information (InfAct) was to reduce health information inequalities between countries.^10^ Within this JA, peer administered HIS assessments were organized because this was expected to provide the ideal setting to exchange good practices and expertise between European countries, which in turn would address these inequalities. Assessors from nine countries performed peer assessments of each other’s national HIS, using an adapted version of the WHO Support tool.^10^ Although peer review is a well-known approach in other comparative settings such as the European co-operation for Accreditation,^11^ it is not a commonly applied approach in HIS assessments.

Therefore, the aim of this study is to evaluate the advantages and disadvantages of the InfAct peer assessment methodology as well as the different steps involved in this assessment process.

## Methods

### Study setting

**Figure 1:**
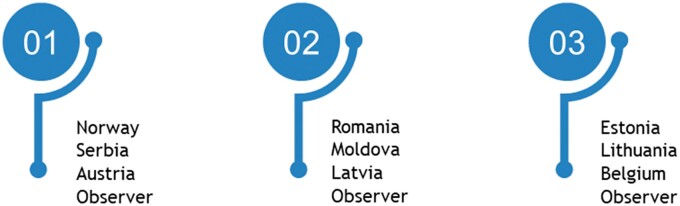
Groups in InfAct Health Information System peer assessments

Assessments were held in Austria, Belgium, Estonia, Latvia, Lithuania, Moldova, Norway, Romania and Serbia. The countries were split into three groups of three countries as shown in figure 1. The peer assessments were carried out in three cycles. The first assessment in each group took place in the period February to March 2019, the second in May to June 2019 and the third in October to November 2019.

### Assessment approach

The assessments were carried out by one or two peer assessors from each assessing country, meaning a maximum of four assessors in total. All assessors were trained in a 2-day course on how to perform the assessment. A contact person in the assessed country acted as the national liaison during the assessment and organized the peer assessment. The contact person is called the host assessor. A single observer provided support during the assessment based on previous experience with the assessment methodology, to ensure that the assessments were performed according to professional standards and procedures.

Each assessment included a preparatory desk report, a country visit with semi-structured face-to-face interviews with local stakeholders, a final report and a follow-up stakeholder meeting. An HIS Assessment Manual was developed providing the objectives, process and guidelines, and roles and tasks related to the InfAct peer review assessment approach.^12^

The host assessor developed a 2-day programme for the country visits. Typical stakeholders included Ministries of Health, National Public Health Institutes, Statistical Offices, and Health Insurance Funds. The assessors carried out the interviews using an HIS items list, covering the following domains: resources, indicators, data sources, data management (including digitalization), national HISs data quality/information products and dissemination and use. Based on the outcomes of the interviews, the assessors wrote a final report, which was presented to the local stakeholders, including those in leadership positions, through a virtual multi-stakeholder follow-up meeting in the assessed country. The participants jointly validated the final reports. The final reports included a SWOT (Strengths, Weaknesses, Opportunities and Threats) analysis, as well as SMART (Specific, Measurable, Assignable, Realistic, Time-related) recommendations for improvement.

### Data collection and analysis

A qualitative evaluation of the peer HIS assessment was performed based on 12 semi-structured interviews. One interview was carried out with the assessors from each country (*N* = 9). Additionally, three interviews were carried out with the observer after each cycle. The interviews were carried out between May 2019 and January 2020. The semi-structured interviews were based on two questionnaires: one for the assessors and one for the observer (Supplementary material). All interviews were carried out by the same person (PB). Interviews were carried out by teleconferencing using GoToMeeting^®^ and were recorded. The duration of the interviews was 1 hour. The interviews were transcribed using Express Scribe Transcription Software^®^. A qualitative content analysis of the 12 interviews was carried out. Common themes across the interviews were identified using a deductive thematic analysis with the following consecutive steps: transcribing and repeated reading of the interviews, extracting of codes, collating codes in broader themes, reviewing themes, defining and naming themes, analyzing the themes in relation to the story that is told and in relation to each other, and reporting themes.^13^ The coding and analysis were carried out with Nvivo 12^®^.

## Results

The results of the evaluation are presented in two sections. Firstly, the experienced advantages and disadvantages of the InfAct peer assessment approach as a whole are presented, and secondly, the evaluation of the specific steps of the assessment methodology is set out.

### Advantages and disadvantages of the InfAct peer assessment approach

#### Advantages

##### Informal atmosphere

One of the advantages of the InfAct peer assessment approach is its informal atmosphere. ‘The atmosphere in the assessments during the actual interviews was quite relaxed’, is said during an interview, ‘as a consequence, during the assessment, stakeholders in the country spoke more openly’. One assessor witnessed: ‘Some of my informers agreed to speak more candidly because they were peers’. Others confirmed the peer assessors were well received on the ground and people opened up easily. People were talking to equals. Another assessor confirms: ‘There was a lot of openness from our government and our Ministry of Health to be part of this process’.

##### Capacity building

Another advantage is that the InfAct peer assessment approach helps building up expertise and knowledge of health information experts from the country, i.e. the assessors participating in the HIS assessment. During an interview it is explained as such: ‘From my point of view, the country benefits from the peer assessment because the capacity is built to carry out their own assessment. Somewhere in the national public health institute two people are trained to do a peer assessment and know the method’. Moreover, experience and knowledge are exchanged between the countries. The assessors learned how their HIS compares to another one. One assessor set it out clearly: ‘We learn from each other. Every time the assessor puts a question forward during the assessment he or she also relates the question to his or her own experience’. Moreover, assessors learn about the full HIS assessment methodology, as participation in all steps of the HIS assessment is required.

##### Objectiveness

Having the assessment done by multiple assessors coming from different countries has a positive impact on the objectiveness of the assessment, as was pointed out by multiple interviewers. ‘It was useful to have more experts in the field. A much larger area of health information could be covered. It is always better to have a bigger pool of knowledge than to have the assessment done by a single person’. Another interviewer said: ‘When you have at least experts from two countries to ask the same question, you have more opportunity to have better questions from different points of view’.

##### Networking

Moreover, the peer assessment approach stimulates the creation of a network within and across countries. As explained during an interview: ‘You create a new identity: a health information community’. The assessments also increase the networking of the assessors: ‘You actually help the assessors to climb a little bit during the assessment because the stakeholder starts looking at them a bit differently during the assessment. It has definitely placed them more on the local map in their health system, not only in their HIS’.

#### Disadvantages

##### Request format

A disadvantage may be that the assessment is not carried out upon a formal request from the Ministry of Health, expressed an interviewee. Therefore, it might be more difficult to get access to some of the stakeholders in the peer review format. However, the assessors were well placed in the HIS according to an interviewee, which allowed them to use their network to engage with stakeholders in the assessment. This was confirmed during multiple interviews such as through this quote: ‘Eventually we all managed to get the right stakeholders on board with very few exceptions’.

##### Setting for recommendations uptake

Another disadvantage is the potential credibility or implementation of the recommendations. An interviewee explained: ‘The challenge is to be taken seriously because people will say: what is this about? Why do I need this? Does this have an impact?’ Therefore, the engagement to take up the recommendation might be lower in a peer assessment approach by not having the weight of an official authoritative institution such as the WHO, reasoned an interviewee. In addition, the stakeholder follow-up meeting was not organized face-to-face in the InfAct peer assessment approach, which might have led to losing the momentum according to an interview.

### The process of the InfAct peer assessment approach

#### The training and HIS assessment manual

A 2-day training was provided to the assessors prior to the assessments. This was appreciated by all the interviewees. During the training, the HIS assessment manual for peer review was explained and participants were split into groups to carry out preparatory exercises. Some examples of experiences include: ‘It was incredibly interesting. It was good to learn about this. It was good to put things in a bigger context. The manual is a good cookbook. Everything was explained’. The manual is very self-explanatory remarked an assessor. ‘You understand what is going on and you understand what you have to do’.

#### The preparatory desk report

The preparation of the preparatory desk report took more time in the first cycle, explained multiple assessors. ‘It was difficult for them to identify what was the most useful information and they tended to go into too much detail. This improved over time’ was mentioned during an interview. The preparatory reports were felt by the assessors to provide a good background on the HIS to be assessed. Language was sometimes an issue when preparing the report, as the information was not always available in English. Moreover, it was questioned by one assessor whether the report should have a formalized structure or not.

#### The country visit with face-to-face interviews

The assessors found the layout of the country visits very satisfactory. Although the assessors indicated that there was a lot of information to cover during the interviews, there was usually enough time for questions. It was pointed out that it was important to explain to the stakeholders before the interviews what an HIS is and what to expect from the assessment. The observer facilitated this by introducing the exercise beforehand.

The assessors sometimes experienced difficulties to engage stakeholders. The most difficult stakeholder to engage was the health insurance fund(s) according to the assessors. However, overall, ‘Those who were well placed in the HIS did not experience difficulties to engage the local stakeholders.’ an interviewer explained. Depending on how familiar the assessors were with the activities of the stakeholder, they experienced difficulties during the actual interviews, according to an interviewee. The following was explained: ‘Those with a wider experience could better exchange with a wider range of stakeholders very actively. Based on their background they may feel more or less comfortable asking certain questions. The wider the background the more capable in carrying out the assessment’.

Additional challenges according to the interviews were the organization of the country visits in 2 days and the identification of the right local stakeholders. Stakeholders had varying levels of knowledge and communication skills. As one assessor put it: ‘Some stakeholders like to talk and talk. Others that were expected to talk did not talk that much. They were asked a question and just said yes or no’. A balance had to be found between responsiveness, interest and competency according to the assessors.

#### The final report

The assessors experienced the final reports to be useful and they appreciated the format of the report as predefined in the HIS Assessment Manual. ‘The structure is excellent. It is very readable. It really responds to what policy makers are willing to read. It is really what they want to know.’ stated an assessor. The assessors said it was not easy to do the SWOT analysis and to prepare the SMART recommendations because you had to be very short and to the point. An assessor witnessed: ‘Every word is weighed against the interest of the different stakeholders. I liked writing the report, it was a very good exercise. I also liked receiving it’. Over time, the assessors explained that they became more practiced at it and recognized it was best to draft the SWOT and recommendation right after the country visit.

#### The stakeholder follow-up meeting

The stakeholder follow-up meeting was the most difficult aspect to organize according to the evaluation interviews. The assessors stated that they struggled to get all stakeholders to participate and engage. The purpose of the meeting was to discuss the final report with the stakeholders and support the uptake of the recommendations; therefore, it was essential for the stakeholders to be present.

#### The organization of the assessment

The assessors appreciated the fact that they were involved in three assessments. For most of them, three assessments were the right number because it was easier to work in smaller groups. Bigger groups would have increased the workload and made communication more complex. An assessor explains why carrying out three assessments was ideal for her: ‘My fear was that two times was enough, because after the second time we understood everything, the whole process. But at the end, after the third cycle, we were completely clear about the steps and the procedures’. The ability to perfect the use of the assessment methodology increased in the last assessment according to the assessors.

#### Overarching remarks

Some assessors indicated that the assessments took more time than expected. Most of the assessors had busy schedules and had difficulties postponing daily activities. ‘Also the two-day country visit was very intense, which allowed us to go in depth in one system and really work on it hard’, as an assessor explains.

The assessors appreciated the presence of the observer providing a positive impact. The observer gave the assessment an official role by starting the meeting and moderating the discussion according to the assessors. ‘He was asking very relevant questions at the right time’ explained an assessor. ‘The observer allowed to share experience and facilitate the discussion’.

The assessors enjoyed the group composition according to the interviews. Some countries in the same group had similar HISs, others had more diverse HISs. Both were perceived as enriching by the assessors. Having similar HIS helped to see how similar issues can be addressed differently and boosted comprehension. An assessor witnessed: ‘The group was well selected because we have the same starting point 20 years ago. It is interesting to see how each country found its own way of development. After that it is very easy and useful to make a comparison’. The disadvantage of having similar HIS was the difficulty to remain objective. As stated during an interview: ‘The secret in choosing the peers, is striking a balance between how close and how far their HIS is from each other’.

## Discussion

### The value of the InfAct peer assessment

The main value of peer HIS assessment approach as carried out in InfAct is its contribution to capacity building in participating countries and its likely reduction of health information inequalities between countries. Initially, it was expected that capacity building would take place by the exchange of best practices. The evaluation suggests that the reduction of health information inequalities may rather have been addressed through the experience and knowledge that was built in the countries during the assessment process. Each step in the assessment process is experienced as essential and brought a different learning experience to the assessors. Each step adds to the capacity building of the assessor. Firstly, the training was important to familiarize the assessors with the HIS assessment tool. Secondly, the preparatory desk report pushed the assessors to synthesize the available information, boosting their reporting skills. Thirdly, the country visits with face-to-face interviews developed their organization and communication skills. The assessors had to organize the country visits in their own country lobbying for participation and engagement of local stakeholders. During the country visits, they had to adapt their way of questioning between the interview which developed their confidence, cultural sensitivity and interview versatility. Fourthly, the final reports developed the assessors’ reporting skills. Fifthly, the stakeholder follow-up meeting developed the assessors’ negotiation skills. They had to incorporate comments of the stakeholders in the final reports, whilst staying true to the information provided in the interviews. Overall, having the peer assessment process in cycles helped the assessors to broaden their understanding of the assessed HISs and their own country HIS, to gain in-depth knowledge on how to carry out an HIS assessment and to repeatedly practice their organization, communication, reporting and negotiation skills. The assessments also created a health information community within and across European countries.

A possible limitation may be that the timing of the interview of the assessors was different, i.e. after each of the three rounds, three assessors were interviewed. Their experience changed along the process. On the other hand, covering the whole period, although with different subjects, provided a more comprehensive overview of the different experiences throughout time.

The advantage regarding the setting, an EU financed project, is that there is an organization coordinating the activity and taking the lead in organizing such an exercise and that these assessments are done among peers from different countries. Moreover, in such a setting the exercise has a bottom-up approach leading to a collaborative effort. On the downside, the exercise is not part of a more permanent strategy, having an impact on sustainability and potentially the uptake of recommendations.

### Recommendations

This study has shown that the InfAct peer assessment methodology is a low-threshold, relatively easy to implement method that thoroughly contributes to capacity building and therefore is likely to address health information inequalities. It allows for structured build-up of knowledge and strengthening of expertise. It has proven to function in diverse European regions and systems. This evaluation confirms its utility and value. Therefore, this study strongly recommends the wide deployment of this methodology and its anchorage in systematic HIS capacity building across European countries. Moreover, the assessments ideally should be integrated in HIS governance strategies both at national and international level for the improvement of the European HIS. Two European projects are already intending to apply the methodology based on the positive experience of InfAct. They will further investigate its use in different settings. The Population Health Information Research Infrastructure will use the peer methodology to assess COVID-19 HISs.^14^ The Joint Action Towards the Health Data Space will investigate how to use the methodology to map the state of play of HISs to connect national systems to a future European Health Data Space.^15^

To strengthen the experience of the assessments and the HIS assessment approach in itself, three additional recommendations emerge from the evaluation. Firstly, the length of the country visits should be extended from 2 to 3 days. This would allow to reduce the intensity of interviews and to organize the final stakeholders meeting directly on the third day. Having this meeting face-to-face on the third day would keep the momentum of interest from the stakeholders and probe the potential uptake of recommendations. Involving the Ministry of Health early on in the process and having the Ministry take ownership of the formulated SMART recommendations may also improve the uptake. Finally, the preparatory report should be prepared in closer collaboration with the host assessor to be a more practical instrument.

In conclusion, the InfAct peer HIS assessment methodology strengthens capacity in national HISs by building up the knowledge and expertise in participating countries and as such addresses health information inequalities. Its main advantages are its informal atmosphere, high level of engagement, high degree of objectiveness and networking opportunities. All five steps of the HIS assessment are essential and each contributes to the learning experience of the participants. This study confirms the value and relatively easy to implement the methodology and therefore recommends its wide and systematic application across Europe.

## Supplementary data

Supplementary data are available at *EURPUB* online.

## Supplementary Material

ckac014_Supplementary_Data
