# Supplementary material for: Capacity building in European health information systems: the InfAct peer assessment methodology
Source: Eur J Public Health. 2022 Mar 23;32(3):463–7. doi: 10.1093/eurpub/ckac014 (PMC9159311; doi:10.1093/eurpub/ckac014)
Supplement: ckac014_Supplementary_Data [file ckac014_supplementary_data.docx]

## SUPPLEMENTARY MATERIAL

**Questionnaires for semi-structured interview**

| **Interview with assessor** |
| --- |
| The participation to this questionnaire is voluntary and you can stop at any time. The interview is being recorded. Would you like to participate to this questionnaire? |
| Section I: focus on content |
| What did you learn during the assessment?  *Sub questions. Each sub question should be covered:*  What did you learn with regards to:   - identification and exchange of good practices - interaction and collaboration between HI experts within and between countries - the understanding of the HIS in own or different country - identification of real strengths, weaknesses, opportunities and threats in the national HIS under assessment and possible recommendations. |
| What has been the impact of the assessment on your work? |
| What happened in your country after the assessment? *(Each sub question should be covered)*   - Impact of exchange of good practices - Impact of knowing each other (network) - Impact of better understanding HIS - Impact of recommendations or SWOTs |
| Section II: focus on process |
| How did you experience the HIS assessment in peer review format and the steps we have gone through? |
| *Sub questions on process. Each sub question should be covered:*  What was your experience with:   - The training in Moldova and manual - The preparation of the preparatory desk report - The country visit with face-to-face interviews - The drafting of the final report - The stakeholder follow-up meeting - Organisation in three cycle |
| When you think of the processes (list elements in sub question above), were there aspects you struggled with or think you dealt well with?  *Things that may come up. They do not all need to be covered:*   - Work-load - Interview difficulties/strengths - Struggles with cultural aspects/language - Preparation (well/not well) - Organisation interviews - Length of assessment |
| *Sub questions on role:*   - How did you experience your role of assessed?   - How prepared did you feel to carry out your role? - How did you experience having two countries and an observer carrying out the assessment? - What did you think of the role of observer? |
| What are the advantages or disadvantages of the peer review format according to you? |
| What do you think of the assessment sheet? |
| How do you think the process of the peer review format could be improved? |
| What do you think are key elements to make a peer review successful? |
| Is there anything else you would like to say? |

| **Interview with observer** |
| --- |
| What was your experience of carrying out the HIS assessment in peer review format? |
| What are the advantages or disadvantages of the peer review format according to you? |
| Sub questions on role: *(Each question should be covered)*   - How did you experience your role of observer? - What do you think are the advantage or disadvantage of having an observer during the assessment? - How prepared did you feel to carry out your role? - How did you experience having two countries and an observer carrying out the assessment? - What were the differences between the groups in the first cycle? |
| How do you think the process of the peer review format could be improved? |
| What do you think are key elements to make a peer review successful? |
| Where there aspects the assessors or assessed struggle with or dealt particularly well?  *Things that may come up. They do not all need to be covered:*   - Work-load - Interview difficulties/strengths - Struggles with cultural aspects/language - Preparation (well/not well) - Organisation interviews - Length of assessment |
| What do you think the participants learned from the assessment? What did you learn?  *Sub questions. Each sub question should be covered:*  What did they learn with regards to:   - identification and exchange of good practices - interaction and collaboration between HI experts within and between countries - the understanding of the HIS in own or different country - identification of real strengths, weaknesses, opportunities and threats in the national HIS under assessment and possible recommendations. |
| Is there anything else you would like to say? |
